# **Biogenic synthesis, characterization, and in vitro biological investigation of silver oxide nanoparticles** (**AgONPs**) **using *Rhynchosia capitata***

**DOI:** 10.1038/s41598-024-60694-3

**Published:** 2024-05-07

**Authors:** Zakir Ullah, Javed Iqbal, Farhat Gul, Banzeer Ahsan Abbasi, Sobia Kanwal, Mohamed Farouk Elsadek, M. Ajmal Ali, Rashid Iqbal, Heba H. Elsalahy, Tariq Mahmood

**Affiliations:** 1 Department of Plant Sciences, Faculty of Biological Sciences, Quaid-i-Azam University Islamabad, Islamabad, 45320 Pakistan; 2 Department of Botany, Bacha Khan University, Charsadda, 24420 Khyber Pakhtunkhwa Pakistan; 3 Department of Botany, Rawalpindi Women University, 6th Road, Satellite Town, Rawalpindi, 46300 Pakistan; 4 Department of Biology and Environmental Sciences, Allama Iqbal Open University, Islamabad, 45320 Pakistan; 5 Department of Biochemistry, College of Science, King Saud University, P.O. 2455, 11451 Riyadh, Saudi Arabia; 6 Department of Botany and Microbiology, College of Science, King Saud University, 11451 Riyadh, Saudi Arabia; 7 Department of Agronomy, Faculty of Agriculture and Environment, The Islamia University of Bahawalpur, Bahawalpur, 63100 Pakistan; 8 Leibniz Centre for Agricultural Landscape Research (ZALF), 15374 Müncheberg, Germany

**Keywords:** *Rhynchosia capitata*, Silver oxide nanoparticles, Cytotoxicity, Anticancer, Biocompatibility, Biochemistry, Plant sciences

## Abstract

The current research aimed to study the green synthesis of silver oxide nanoparticles (AgONPs) using *Rhynchosia capitata* (RC) aqueous extract as a potent reducing and stabilizing agent. The obtained RC-AgONPs were characterized using UV, FT-IR, XRD, DLS, SEM, and EDX to investigate the morphology, size, and elemental composition. The size of the RC-AgONPs was found to be ~ 21.66 nm and an almost uniform distribution was executed by XRD analysis. In vitro studies were performed to reveal biological potential. The AgONPs exhibited efficient DPPH free radical scavenging potential (71.3%), reducing power (63.8 ± 1.77%), and total antioxidant capacity (88.5 ± 4.8%) to estimate their antioxidative power. Antibacterial and antifungal potentials were evaluated using the disc diffusion method against various bacterial and fungal strains, and the zones of inhibition (ZOI) were determined. A brine shrimp cytotoxicity assay was conducted to measure the cytotoxicity potential (LC_50_: 2.26 μg/mL). In addition, biocompatibility tests were performed to evaluate the biocompatible nature of RC-AgONPs using red blood cells, HEK, and VERO cell lines (< 200 μg/mL). An alpha-amylase inhibition assay was carried out with 67.6% inhibition. Moreover, In vitro, anticancer activity was performed against Hep-2 liver cancer cell lines, and an LC_50_ value of 45.94 μg/mL was achieved. Overall, the present study has demonstrated that the utilization of *R. capitata* extract for the biosynthesis of AgONPs offers a cost-effective, eco-friendly, and forthright alternative to traditional approaches for silver nanoparticle synthesis. The RC-AgONPs obtained exhibited significant bioactive properties, positioning them as promising candidates for diverse applications in the spheres of medicine and beyond.

## Introduction

Nanotechnology, focusing on synthesizing, modifying, and characterizing nanoparticles sized 1–100 nm, is a rapidly growing research domain^1,2^. Nanoparticles possess unique properties with broad applications in biomedicine, drug delivery, cosmetics, electronics, optics, energy, and bioremediation, attracting global research interest^3,4^. Several techniques exist to produce nanoparticles, involving chemical, physical, and biological approaches, each given its own set of advantages and limitations^5^. Among these approaches, the biological synthesis of nanoparticles offers notable advantages, particularly due to its simplicity, environmental friendliness, and elimination of the need for toxic and expensive chemicals to reduce, stabilize, and coat precursor salts^6,7^. This biologically determined and cost-effective green synthesis has gained expertise among researchers, making it a particularly desirable approach^8^. Consequently, there is a growing interest in utilizing biological methods for sustainable and eco-friendly nanoparticle production^9,10^.

The biological synthesis of nanoparticles incorporates the utilization of diverse biological resources, including bacteria, fungi, algae, plants, and their cellular constituents^11^. However, the application of microbes, such as bacteria and fungi, in nanoparticle synthesis has prompted biosafety concerns, thus limiting their synthesis and broader applications^12^. In contrast, the use of medicinal plants for nanoparticle synthesis is a preferred approach due to the presence of a rich array of biologically and pharmacologically active compounds. These include alkaloids, flavonoids, carboxylic acids, terpenoids, aldehydes, ketones, amides, and ascorbic acids, which play pivotal roles in the reduction and stabilization of metal precursors^13^. Thus, various types of nanoparticles (NPs) have been successfully synthesized through this method^14^. Metal nanoparticles (MNPs) have gained heightened interest for their diverse applications, owing to their promising biological and physicochemical attributes^15,16^. Among these MNPs, silver oxide nanoparticles (AgONPs) have emerged as prominent candidates due to their distinctive properties, which encompass significant antibacterial and antifungal activities, along with noteworthy anticancer potential. As a result, AgONPs find extensive utility in various commercial sectors, including biomedicine, food industries, and household products^8,17,18^.

In a recent investigation, AgONPs were synthesized using an extract derived from the medicinal plant *R. capitata*, which functioned as a potent reducing and capping agent. *Rhynchosia capitata* is esteemed for its multifaceted therapeutic characteristics, attributable to an array of isolated compounds, encompassing flavonoids, isoflavonoids, flavan-3-ols, xanthones, biphenyls, simple polyphenols, sterols, C-glycosylflavonoids, and prenylated isoflavonoids. Its historical utilization in the management of diverse ailments such as intestinal helminthiasis, dysentery, diarrhea, polymenorrhea, anemia, ulcerations, menorrhagia, hypocholesterolemia, thyroxine-induced hyperglycemia, and diarrheal conditions underlines its medicinal significance^19,20^. Furthermore, the study's objectives covered the comprehensive assessment of various biological activities associated with the synthesized AgONPs. To the best of our knowledge and based on an extensive review of the existing literature, this study represents the pioneering endeavour in the orbit of green synthesis techniques, employing leaf extracts of *R. capitata* to produce AgONPs.

## Material and methods

### Plant collection and synthesis of extract

The medicinal plant *R. capitata* (Leguminosae) was collected from Margalla Hills Islamabad, Pakistan following established protocol and permission was obtained. Our plant study complies with relevant institutional, national, and international guidelines and legislation. The collected plant samples were taxonomically authenticated and identified by a taxonomist at https://qau.edu.pk with authorization number: SAS-561. The leaves of RC were carefully removed thoroughly cleaned with distilled water, and shade dried for approximately 5 weeks. Furthermore, employing an earlier protocol^21^ the sample was crushed to a light powder and kept in an airtight container to prevent direct sunlight. Then, 400 mL of distilled water was added to 40 g of dry *R. capitata* powder and heated to 70 °C for 3 h. The subsequent solution was cooled to normal temperature and filtered three times using filter papers to obtain a pure aqueous extract.

### Synthesis of RC@AgONPs

**Figure 1:**
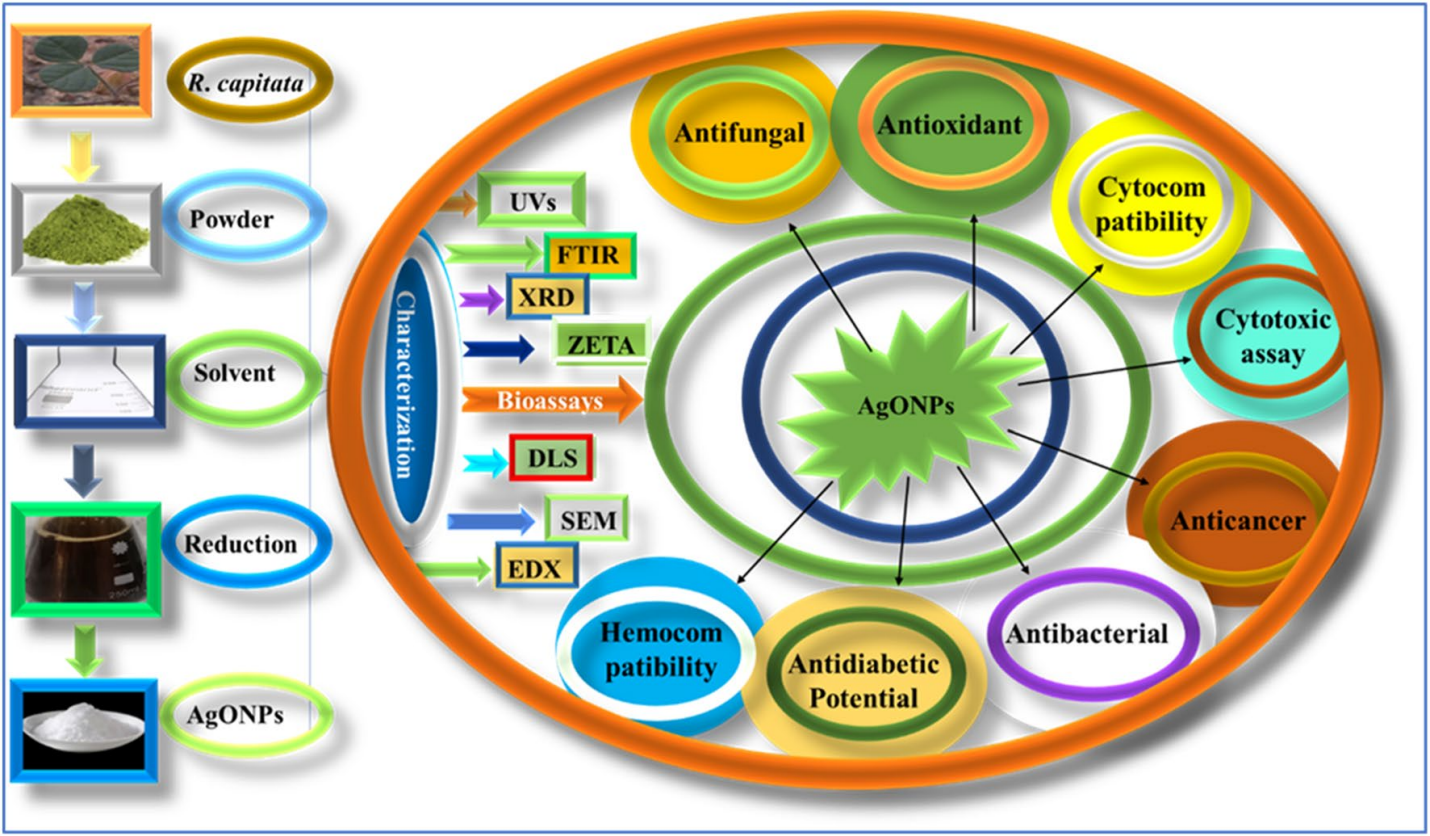
The schematic diagram shows *R. capitata*-mediated green synthesis, characterization, and biological activities of AgONPs.

The synthesis of nanoparticles was achieved utilizing a previously optimized protocol^21^. The reaction mixture was prepared by the addition of 100 mL of filtered *R. capitata*-mediated extract with 3 g of silver nitrate (AgNO_3_) (Sigma Aldrich, Saint Louis, MO, USA) salt and was heated at 70 °C for 3 h. The resultant mixture was centrifuged at 3000 rpm for 30 min. Furthermore, the subsequent powder, considered to be AgONPs, was placed in a furnace for 60 min at 500 °C, and the obtained powder was carefully collected and stored for further use. Additionally, the synthesized AgONPs were extensively checked and characterized by employing respective analytical tools. Finally, the synthesized nanoparticles were evaluated to investigate several biological activities (Fig. 1).

### Characterization approaches of RC@AgONPs

The preparation of AgONPs and other physicochemical properties were determined and confirmed by using various characterization techniques^22^. The variation in color in the silver nitrate solution incubated with aqueous plant extract indicated the synthesis of AgONPs. However, the solution of AgONPs was scanned via a UV-4000 UV‒Vis spectrophotometer (Germany) in a range of 200–800 nm to confirm the exact absorption peak. Furthermore, FT-IR analysis was performed to examine various functional groups involved in reducing, stabilizing, and capping metal precursors. Moreover, using a scanning electron microscope (SEM), the test sample was scrutinized between 4500 and 450 cm^−1^. This investigation was performed to determine the shape and topology of the synthesized AgONPs. An EDX assessment was performed to confirm the elemental constituents and composition of AgONPs. Also, XRD testing was performed to determine the crystalline nature of the synthesized AgONPs. The Scherrer equation was applied to compute the corresponding size of the samples after examination with an X-ray diffractometer. Moreover, the crystal clarity and particle size were evaluated using a PANalytical Empyrean Diffractometer. The polydispersity index (PDI), hydrodynamic size distribution, and surface charge of the nanoparticles were inspected using a DLS system and a Malvern Zetasizer (Nano S90).

### RC@AgONPs therapeutic capacity

#### Brine shrimp cytotoxicity assessment

Brine shrimp cytotoxicity analysis (BSCA) was performed to investigate the cytotoxicity potential (CP) of AgONPs^23^. To determine the CP of AgONPs, *Artemia salina* eggs were cultured in the presence of light in artificial seawater for 24 h at 27 °C. After 24 h of incubation, 15 nauplii were transferred to glass vials, and their dose-dependent response was studied using different concentrations of AgONPs (37.5–1000 μg/mL). Vials containing vincristine sulfate were used as a positive control, whereas glass vials containing DMSO were considered negative controls. Additionally, the vials were incubated in an incubator for 24 h at 30 °C. The number of living and dead shrimp in each vial was carefully counted after 24 h. GraphPad software version 8.0.0 was used to calculate the IC_50_ values for AgONPs.

#### Alpha-amylase inhibition potential of RC@AgONPs

The antidiabetic potential of RC@AgONPs was evaluated using an alpha-amylase inhibition assay^21,24^. Alpha-amylase is an enzyme found in the pancreas and saliva of humans and other mammals and is responsible for digesting carbohydrates. The reaction mixture was prepared by combining the succeeding components in a 96-well microplate: 40 μL of starch solution, 25 μL of alpha-amylase, 15 µL phosphate buffer saline with pH 6.8, and 30 μL of AgONPs. The microplate with the mixture was then incubated for 90 min at a temperature of 50 °C. After that, the reaction mixture was supplemented with iodine solution (90 µL) and 1 M hydrochloric acid (20 µL). It can be assumed that the addition of iodine solution and HCl at this point is part of a timed reaction protocol or a specific experimental design. The overall purpose of this reaction mixture and subsequent additions of iodine and HCl might be to study the effect of AgONPs and alpha amylase on the breakdown of starch. Acarbose and DMSO were used as positive and negative controls, respectively. Furthermore, the microplate was examined by using a microplate reader, and the optical density (O.D.) was measured by scanning the reaction mixture at a wavelength of 540 nm. The median lethal concentration (LC_50_) value was calculated employing GraphPad Prism (8.0.0), and % inhibition was checked by using the formula provided below:

$$\% {\text{Inhibition}} = \left( {{\text{Abs}}\;{\text{of}}\;{\text{sample}}{-}{\text{Abs}}\;{\text{of}}\,\, - {\text{ve}}\,{\text{control}}/{\text{Abs}}\;{\text{of}}\;{\text{blank}}{-}{\text{Abs}}\;{\text{of}}\, - {\text{ve}}\;{\text{control}}} \right) \times {1}00$$

#### Antibacterial potential of RC@AgONPs

In recent research, the assessment of the antibacterial activity of RC@AgONPs via disc diffusion methodology against various bacterial strains was conducted^25^. To perform the experiment, bacterial strains were subcultured in a nutrient broth medium overnight at 37 °C and were streaked with a cotton swab on nutrient agar media to accomplish a uniform bacterial culture. Furthermore, the bacteria-loaded Petri dishes were treated with different concentrations (75–1000 μg/mL) of RC@AgONPs to evaluate the dose-dependent response. After 24 h of incubation, the zone of inhibition was observed and measured. Finally, MIC values were calculated to determine the dose-dependent response of RC@AgONPs. In this experiment, oxytetracycline, an antibiotic, was employed as a positive control to assess the expected response in the test system. A negative control was established using 5% DMSO, which served as the solvent for the experimental compounds.

#### Antifungal potential of RC@AgONPs

The antifungal activity of AgONPs-mediated *R. capitate* was examined using a Sabouraud dextrose (SDA) liquid medium following the disc diffusion method^26^. Numerous fungal strains were employed to assess the antifungal activity. Prior to the fungicidal test, fungal spores were subcultured in SDA media and incubated overnight at 37 °C. The optical density (O.D.) of the fungal strains was calibrated to 0.5. Furthermore, different AgONPs doses (75–1000 µg/mL) were employed against various fungal strains and again incubated at 37 °C. Experimental Outcomes were evaluated with Amp-B and DMSO as positive and negative controls, respectively. Their zones of inhibition (ZOI) values were recorded and calculated after 48 h of incubation to verify their antifungal capabilities.

#### Hemocompatibility assay

A biocompatibility test was carried out to determine whether AgONPs were physiologically appropriate for human erythrocytes (RBCs)^27^. To prepare the reaction mixture for the biocompatibility experiment, 90 µL of freshly acquired human red blood cells (RBCs) were carefully added to a tube containing EDTA as an anticoagulant. The tube was then subjected to centrifugation at 7000 rpm for 10 min. The centrifugation process effectively separates the red blood cells from the plasma and allows for the isolation of the RBCs required for the subsequent steps of the biocompatibility experiment. The resultant pellet (2–3 times) was washed with phosphate-buffered saline at pH 7.5. Furthermore, 200 µL of RBCs and 9.8 mL of PBS buffer were mixed to produce erythrocyte suspensions. After that, the erythrocyte solution (100 µL) was added to various AgONPs dosages ranging from 17 to 1000 µg/mL and was allowed to incubate at 37 °C for 1–2 h before being passed through a centrifuge at 13,000 rpm for 25 min. Likewise, the supernatant was added to a 96-well plate, whereby the quantity of haemoglobin shattering was calculated and quantified by using an Eliza microplate reader at 540 nm. Triton X-100 was retained as a positive control, while DMSO was utilized as a negative control. The formula of % hemolysis was used to compute the outcomes.

$${\text{Percent}}\;{\text{Hemolysis}} = \left( {{\text{Abs}}\;{\text{of}}\;{\text{sample}}{-}{\text{Abs}}\;{\text{of}}\;{\text{negative}}\;{\text{control}}} \right)/\left( {{\text{Abs}}\;{\text{of}}\;{\text{negative}}\;{\text{control}}} \right) \times {1}00$$

#### RC@AgONPs -mediated antioxidant assay

The free radical scavenging capability of AgONPs was examined via spectrophotometry^28^. The reaction mixture for the antioxidant potential was prepared by the addition of 2.4 mg of 2,2-diphenyl-1-picrylhydrazylhydrate (DPPH) in 25 mL of methanol. Various concentrations of AgONPs (37–1000 µg/mL) were used to check their free radical scavenging potential. Moreover, the test sample was prepared in 96-well plates by adding 20 µL of AgONPs to 180 µL of reagent solution and was incubated for one hour in dark conditions. The positive control was ascorbic acid (AA), while DMSO was used as a negative control. Using the formula below, a microplate reader was used to scan and determine their antioxidant potential at 517 nm.

$${\text{DPPH}}\;{\text{scavenging}} = {1} - \left( {\left( {{\text{Abs}}\;{\text{of}}\;{\text{sample}}} \right)/\left( {{\text{Abs}}\;{\text{of}}\;{\text{control}}} \right)} \right) \times {1}00$$

Similarly, the total reducing power of AgONPs was estimated using the previously described optimized potassium-ferricyanide method^21,28^. The optical density of the reaction mixture was determined via an Eliza reader at 630 nm. The power reduction of nanoparticles was represented by grams of ascorbic acid equivalent/mg. Moreover, the antioxidant capacity of biological test samples was measured by total antioxidant capacity (TAC). Optical density was estimated by employing a microplate reader at 695 nm wavelength.

#### Cytocompatibility potential of RC@AgONPs

The cytotoxicity and cytocompatibility of the synthesized AgONPs were tested using VERO (renal epithelial cells were taken from an African green monkey (*Chlorocebus* sp) and HEK cell lines (HEK 293, human embryonic kidney cells)^29^. The cell lines were acquired from the National Institutes of Health (NIH) https://www.nih.org.pk Islamabad. Approximately 1 × 10^4^ cells (10,000 total cells) were placed in each well of a 96-well plate. Different AgONPs concentrations ranging from 5 to 100 μg/mL were used to reveal the dose-dependent response. The treated cells were placed in a CO_2_ incubator at 37 °C for 24 h to allow for their growth and response to the treatment. After the incubation period, each well in the microplate was supplemented with 10 μL of MTT (3-(4,5-dimethylthiazol-2-yl)-2,5-diphenyltetrazolium bromide) solution at a concentration of 5 mg/mL. The cells were then cultured for an additional 3 h to facilitate the formation of formazan crystals through the reduction of MTT by viable cells. Following the 3-h incubation, the formazan crystals were dissolved in approximately 100 μL of dimethyl sulfoxide (DMSO), and the mixture was further incubated for approximately 50 min. This step ensures that the formazan crystals are fully dissolved, and the resulting solution becomes suitable for absorbance measurement. To assess cell viability, the absorbance value of each well was determined at a wavelength of 570 nm using an ELISA plate reader. By employing the cell inhibition formula, the percentage of cell viability was then calculated.

$${\text{Cell}}\;{\text{inhibition}} = \left( {{\text{O}}.{\text{D}}.\;{\text{of}}\;{\text{treated}}\;{\text{cells}}/{\text{O}}.{\text{D}}.\;{\text{of}}\;{\text{control}}} \right) \times {1}00$$

The following formula was used to determine cell viability: (100–cell inhibition).

#### Anticancer assay of RC@AgONPs

The anticancer potential of RC@AgONPs was tested by treating the Hep-2 liver cancer cell line^30^. The cell lines were grown in DMEM. To achieve this purpose, each 96-well plate was seeded with ~ 1 × 10^4^ cells (10,000 total cells) and checked under a microscope. In the subsequent step, Hep-2 cells were subjected to various doses of AgONPs, ranging from 5 μg/mL to 100 μg/mL. These treated cells were then incubated in a CO_2_ incubator at a constant temperature of 37 °C for a duration of 24 h to allow the AgONPs to interact with the cells and induce their effects. After the 24-h incubation period, the DMEM in each well was carefully removed, and fresh 10 μL of MTT solution at a concentration of 5 mg/mL was added to each well. The cells were further cultured for approximately 3 h to enable the conversion of MTT into formazan crystals, which is a metabolic indicator of viable cells. Next, the formazan crystals were dissolved by adding approximately 100 μL of DMSO solvent to each well. Finally, the absorbance value of each well was measured at a wavelength of 570 nm using an ELISA plate reader. By using the following formula:

$$\% {\text{Cell}}\;{\text{Viability}} = \left( {{\text{mean}}\;{\text{absorbance}}\;{\text{of}}\;{\text{test}}\;{\text{compound}}} \right)/\left( {{\text{mean}}\;{\text{absorbance}}\;{\text{at}}\;{\text{control}} - {\text{untreated}}\;{\text{cells}}} \right) \times {1}00$$

### Ethics approval and consent to participate

This study does not include human or animal subjects.

## Results and discussion

### Processing and synthesis of RC@AgONPs

In the current study the leaf extract of *R. capitata*, a widely recognized medicinal plant renowned for its therapeutic attributes, as a pivotal stabilizing and bioreductant agent was used for the efficient synthesis of silver oxide nanoparticles (AgONPs). Biomolecules were screened out to analyse their presence in the extracts of *R. capitata* (Table 1). Employing an array of characterization techniques, the study elucidated the inherent properties and structural attributes of the newly synthesized AgONPs. These findings not only serve as a foundation for more in-depth exploration but also open avenues for prospective research endeavours focusing on the biomedical utility of these biocompatible nanoparticles.

**Table 1:** Phytochemical analysis of *R*. *capitata*.

S/No	Secondary metabolites	
1	Phenol	+ + +
2	Flavonoids	+ + +
3	Alkaloids	+ + +
4	Terpenoids	+ + +
5	Saponins	+ + +
6	Tannins	+ + +
7	Steroids	+ + +
8	Glycosides	−
9	Phytosterol	+ +
10	Coumarins	+
11	Proteins	+ +
12	Carbohydrates	+ + +
13	Anthocyanin&	−
	BetaCyanin	
14	Phlobatannins	−
15	Quinone	+ +
16	Fats and Fixed Oils	+ + +

### UV–Vis spectrophotometry of RC@AgONPs

**Figure 2:**
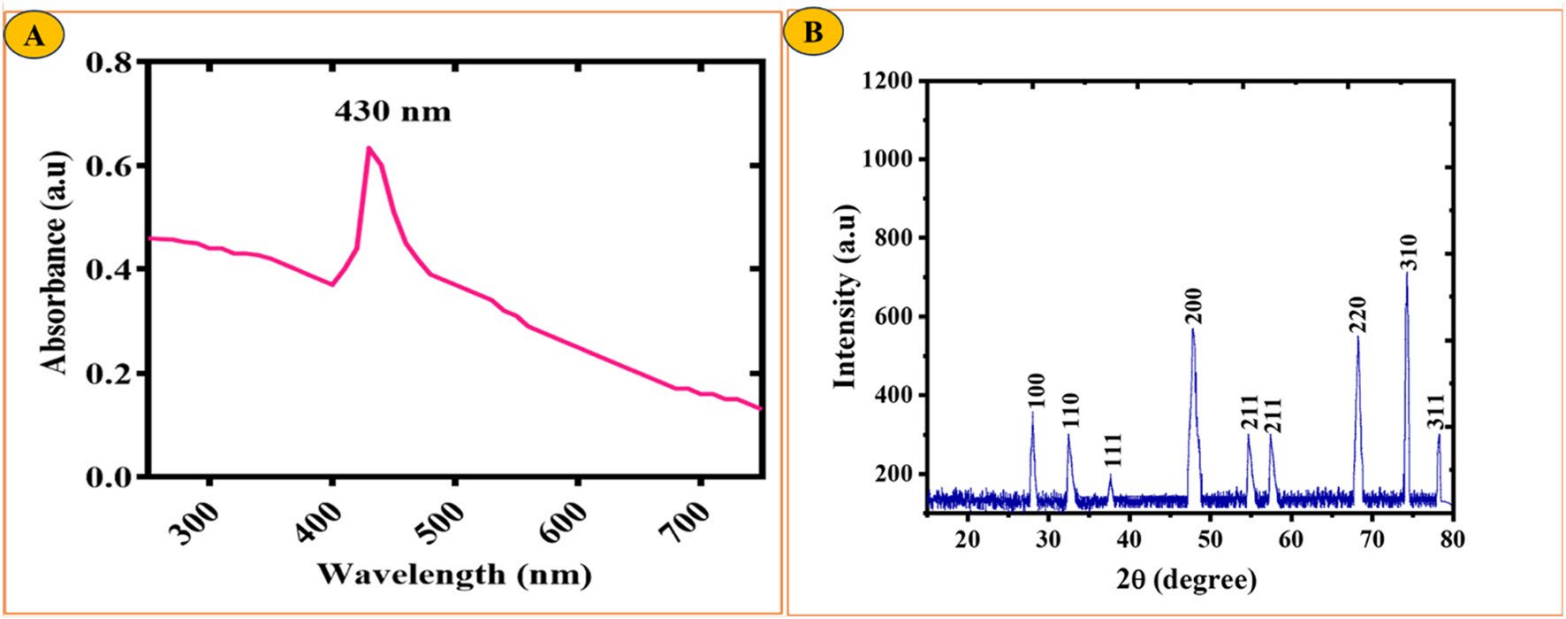
(**A**) The UV visible spectrum of biologically synthesized AgONPs (**B**) XRD spectra of* R*. *capitata*-mediated AgONPs.

In this study, the successful reduction process of the precursor solution was indicated by a noticeable color variation upon the addition of an aqueous extract. The change in color from light brown to a darker brown hue suggested increased bioreduction, indicating the formation of AgONPs. To further confirm the production of AgONPs, the researchers employed a UV–Vis spectrophotometer in the wavelength range of 200–800 nm. The obtained UV–Vis spectra showed a characteristic absorbance peak for AgONPs at approximately 430 nm, which falls within the surface plasmon resonance (SPR) range specific to AgONPs. This absorbance peak at the mentioned wavelength in Fig. 2A provided strong evidence for the successful synthesis of AgONPs in the reaction mixture. The findings of this study, determined by UV–Vis spectroscopy, align with previous investigations that also utilized various types of plant extracts for the synthesis of nanoparticles^31^. This consistency in the outcomes reinforces the credibility of the current research and underscores the effectiveness of using plant extracts as a green and eco-friendly approach for the synthesis of biocompatible nanoparticles, such as AgONPs^32,33^.

### X-ray diffraction spectroscopy of RC@AgONPs

The crystallinity of AgONPs was monitored using X-ray diffraction spectroscopy (XRD) analysis. Figure 2B shows the XRD peaks of AgONPs via biological synthesis. The crystalline reflections from JCPDS pattern 00-076-1393 were found to be reflected at angles of 100 (27.43°), 110 (31.98°), 111 (46.89°), 200 (48.11°), 211 (54.3°), 211 (57.04°), 220 (68.72°), 310 (72.84°), and 311 (78.14°), and it was found that the subsequent Bragg peaks matched these patterns of reflections. The typical size of the AgONPs was determined to be 21.66 nm by employing Debye Scherrer's equation (D = k/12 cos). These results are in line with the earlier findings reported by^34,35^. The findings of the XRD study of biogenic AgONPs are shown in Fig. 2B. AgO was found to have a single, pure phase that matched the reported Bragg peaks (JCPD #: 00-076-1393). The fact that there are no Bragg peaks for other chemical compounds shows that biogenic AgONPs are completely pure and crystalline with a uniform structure^36^.

### Fourier transform infrared spectroscopy (FTIR) of RC@AgONPs

**Figure 3:**
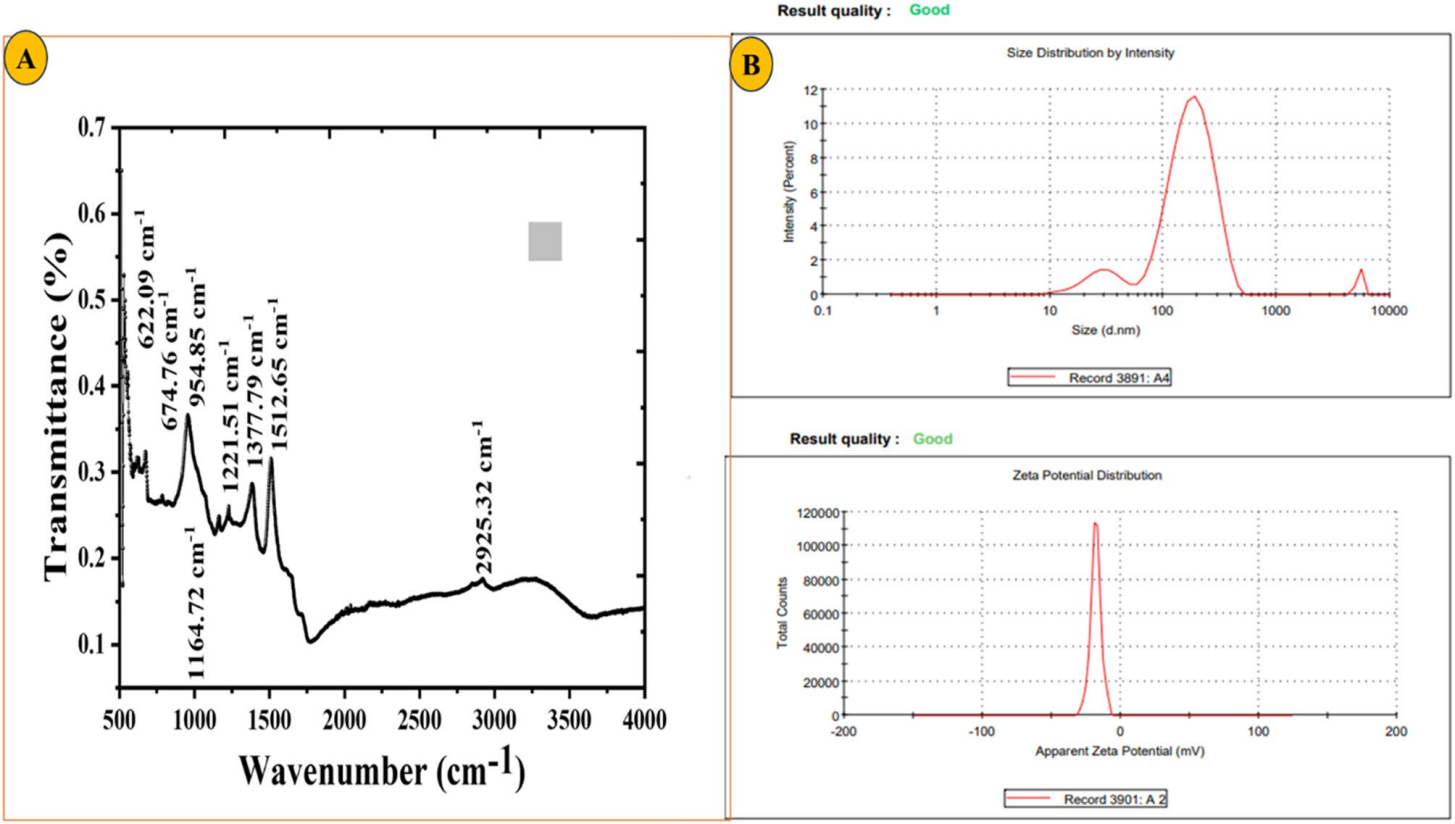
(**A**) FT-IR spectra of biogenic AgONPs using *R*. *capitata* (**B**) The size distribution of AgONPs, The measurement of the zeta potential of AgONPs.

In this study, FT-IR spectra were utilized to analyse the molecular vibrations, presence of biomolecules, and functional groups to evaluate the efficacy of the synthesis and stabilization of AgONPs. Figure 3A displays the FT-IR analysis for AgONPs, and several characteristic bands were observed at specific wavenumbers, indicating the presence of various functional groups. At 674.76 cm^−1^, bands corresponding to C–Cl stretching (a halo compound), C=C bending (alkenes), and C–Br stretching (another halo compound) were identified. Additionally, the bands at 1164.72 cm^−1^ indicated the stretching of C–O bonds, while the bands at 954.85 cm^−1^ represented C-H bending vibrations^37^. Furthermore, the bands at 1221.51 cm^−1^ indicated the stretching vibrations of C–O in alkyl and aryl ether functional groups. At 1377.79 cm^−1^, signals were detected for C–F stretching and O–H bending (alcohol), and at 1512.65 cm^−1^, signals were detected for N–O stretching (nitro compound)^21^. Moreover, a significant signal at 2925.32 cm^−1^ suggested the stretching vibrations of O–H, C–H, and N–H bonds. Additionally, C–Cl stretching vibrations in the halo compound were evident in the bands observed at 622.09 cm^−1^^34^. Table 2 provides a summary of these characteristic bands and their respective assignments, aiding in the interpretation and understanding of the FT-IR spectra results for AgONPs. These results are in line with the earlier findings reported by^21,31,32,34,37^. Overall, the FT-IR analysis offered valuable insights into the molecular structure and involvement of functional groups in the synthesis and stabilization of AgONPs, contributing to the comprehensive characterization of these nanoparticles.

**Table 2:** Functional groups associated with the R.C*@* AgONPs surface area.

S. no	Wavenumber (cm^−1^)	Functional groups
1	2925.32 cm^−1^	O–H/C–H/N–H stretching of amines and amides
2	1512.65 cm^−1^	N–O stretching nitro compound
3	1377.79 cm^−1^	C–F stretching, O–H Bending (alcohol)
4	1221.51 cm^−1^	C–O alkyl aryl ether
5	1164.72 cm^−1^	C–O stretching, C–F stretching
6	954.85 cm^−1^	C–H bend
7	674.76 cm^−1^	C–Cl stretching (halo compound), C=C bending (alkenes), C–Br stretching (halo compound)
8	622.09 cm^−1^	C–Cl stretching halo compound

### Zeta Potential of RC@AgONPs

Zeta potential and DLS approaches were used to evaluate the size and charge of *R. capitata*-mediated AgONPs. Due to high phytochemical adsorption on the surface of the produced NPs, the AgONPs exhibited a negative charge zeta potential^38^. They also made the particles more stable and prevented them from clumping^37^. The polydispersity index (PDI) was 0.428, as shown in Fig. 3B. Low PDI values indicated high-quality, polydisperse particles. The characteristics of these NPs made them ideal for biological assays^33^. NPs' zeta potential levels normally have to be between + 30 mV and − 30 mV to be considered stable. AgONPs were found to have a Zeta value of − 18.1 mV (Fig. 3B). The findings of earlier studies using plant extract-mediated AgONPs are consistent with our present work using *R. capitata*-mediated AgONPs^31,34,37^.

### Scanning Electron Microscopy of RC@AgONPs

**Figure 4:**
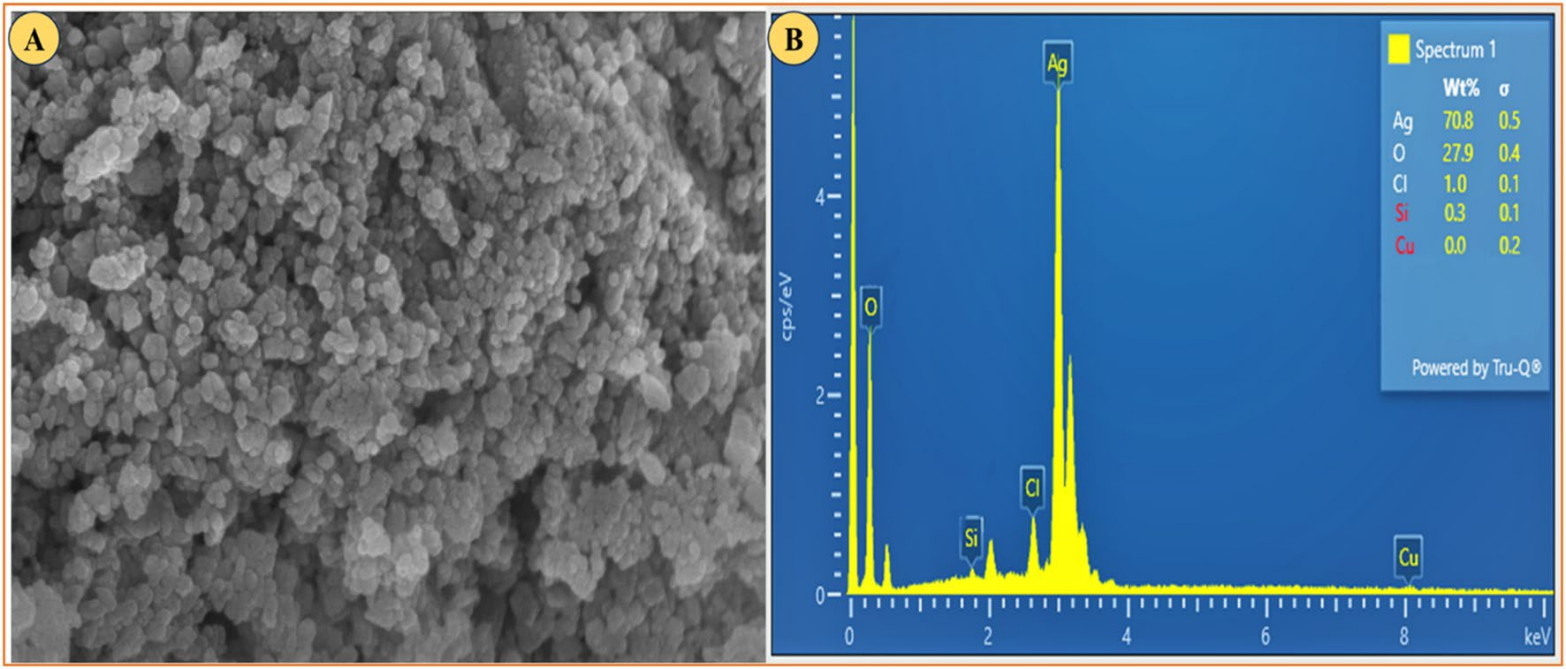
(**A**) SEM review of biogenic *R*. *capitata-*mediated AgONPs (**B**) Elemental composition of AgONPs using EDX.

The morphological aspects of the synthesized NPs were scanned and imaged using SEM analysis. To achieve this purpose, a tiny amount of RC@AgONPs was deposited on a copper grid that was dried with a hand drier to remove any additional particles. This established grid was used to measure the dimensions and shape of the RC@AgONPs under a scanning electron microscope. Figure 4A displays SEM images of AgONPs produced by *R. capitata* and demonstrates that the produced NPs have a round spherical shape^31,35,39^.

### Energy-dispersive X-ray spectroscopy (EDX) of RC@AgONPs

EDX was used to examine the elemental composition of AgONPs. The significant signals for oxygen and silver are in Fig. 4B illustrates the purity of the produced AgONPs. The single-phase purity of the NPs is only connected with the main components "Ag" and "O", and their strong signals (70.8% Ag and 27.9% O) were observed between 0.1 and 3 keV. In addition, some friction of Cl, Si, and Cu (1.0, 0.3, and 0.0%) respectively were also present. Typically, metallic silver nanocrystals exhibit a characteristic optical absorption peak around 3 keV, which arises from their surface plasmon resonance phenomenon. Additional peaks may be attributed to the presence of biomolecules on the surface of the silver nanoparticles. Various substances, including flavonoids, isoflavonoids, biphenyls, flavan-3-ols, xanthones, simple polyphenols, and sterols, have thus far been identified from the genus Rhynchosia^29^. These chemicals play a crucial role in stabilizing NPs by adhering to metal ion surfaces^36,39,40^. They can be found in the *R. capitata* aqueous extract.

#### *Brine-shrimp cytotoxicity *(BSC)* test of RC@AgONPs*

**Figure 5:**
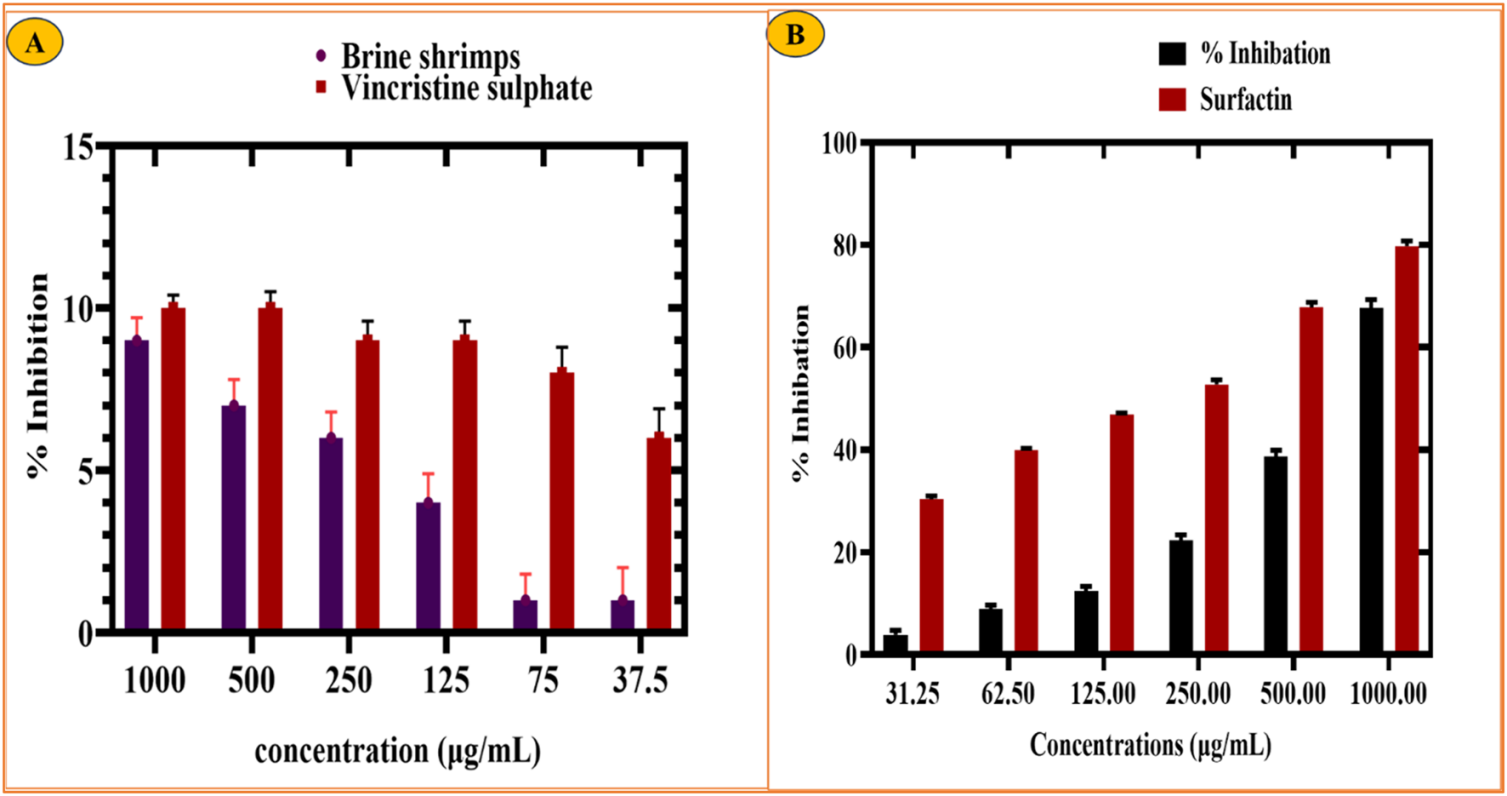
(**A**) Cytotoxic efficiency of *R*. *capitata*-mediated AgONPs on brine shrimps (**B**) Antidiabetic ability of AgONPs against an enzyme (*alpha*-*amylase*).

A brine shrimp cytotoxic assay^30^ was performed to evaluate any naturally occurring compound’s potential. The cytotoxic potential of AgONPs was determined in a dose-dependent manner, ranging from 1000 to 37.5 μg/mL. The BSC potential was recorded as 90% at the highest dose (1000 μg/mL) with an LC_50_ value of 2.26 µg/mL. The cytotoxic abilities rose as the concentration of NPs increased (Fig. 5A). Our findings on RC@AgONPs are correlated with earlier research on AgONPs employing *Parieteria alsinaefolia Delile*, *Simarouba glauca*, *Celastrus paniculatus*, and *Rhamnus virgata*^21,41,42^. These findings supported the potential of AgONPs to trigger cytotoxicity. Nevertheless, not all RC@AgONPs doses showed a greater % inhibition than vincristine sulfate, with an LC_50_ (1.976 µg/mL) utilized as a positive control.

#### *Alpha-amylase inhibition *(AA)* of RC@AgONPs*

The alpha-amylase inhibition potential^43^ of RC@AgONPs was investigated. The current biological assay aims to determine the antidiabetic potential of AgONPs. To do this, AgONPs were used in ranges from 31.25 to 1000 μg/mL. The rate of inhibition steadily decreases as AgONPs concentrations drop. Maximum inhibition (67%) was seen at 1000 μg/mL, 38.6% at 500 μg/mL, and showed decreased outcomes of 22.3% at 250 μg/mL. However, not all the AgONPs dosages studied offered an inhibitory percentage higher than that of the surfactant (positive control). Since the alpha amylase enzyme converts carbohydrates into glucose molecules^34^, inhibiting its potential may reduce the sugar level of the blood, which is a vital area of study on diabetes^44^. The successful ability of AgONPs to suppress alpha amylase can be observed in (Fig. 5B). Our results of green AgONPs agree with earlier studies^45,46^.

#### Antibacterial activity of RC@AgONPs

**Figure 6:**
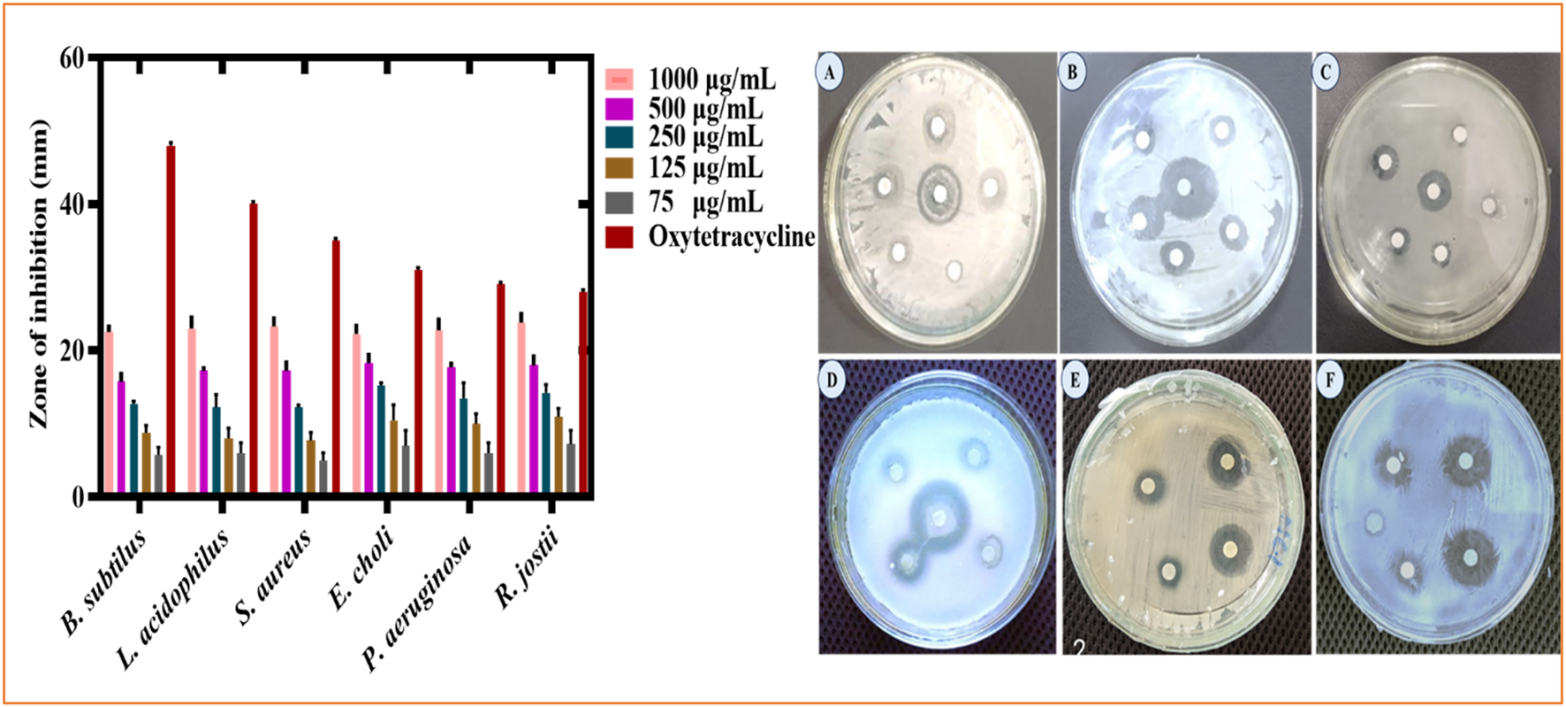
Antibacterial potential of *R*. *capitata*-mediated AgONPs at different concentrations.

In the current study, the antibacterial potency of silver nanoparticles was evaluated. Biogenic AgONPs were checked for their antibacterial activity^47^ against several bacterial strains with doses ranging from the highest 1000 to the lowest 75 µg/mL. *Lactobacillus acidophilus* (ATCC 4356), *Staphylococcus aureus* (ATCC 23,235), *R*. *josti*, and *Bacillus subtilis* (ATCC 23,857) were utilized as gram-positive bacterial strains, while *Escherichia coli* (ATCC BAA-2471) and *Pseudomonas aeruginosa* (ATCC15442) were gram-negative bacterial strains. At various concentrations, AgONPs had a significant impact on bacterial strains (Fig. 6).

According to our studies, the bacterial strain (*S*. *aureus*) was susceptible to AgONPs at the highest applied concentration, i.e., 1000 µg/mL, and manifested a ZOI of 23.25 mm and a ZOI of 6.5 mm at 75 μg/mL. Likewise, *Lactobacillus acidophilus* revealed 23 mm ZOIs at a concentration of 1000 µg/mL and 7 mm ZOIs at a concentration of 75 µg/mL. Likewise, at 1000 μg/ml, *E*. *coli* showed a 22.25 mm ZOI, while it was 8.5 mm at a concentration of 75 µg/mL. Additionally, *P*. *aeruginosa* showed a ZOI of 22.75 mm at a concentration of 1000 µg/mL and 6.85 mm at the lowest applied concentration of 75 µg/mL. In the same way, strain *Bacillus subtilis* was somehow observed to be susceptible to AgONPs, and its ZOI was reported to be 22.5 mm at 1000 μg/ml, while *R*. *jostii* was vulnerable at 1000 µg/mL (ZOI was 23.8 mm). Details about ZOI values are listed in Table 3. An antibiotic (oxytetracycline) was used as a positive control and was shown to be more efficient than any individual sample of AgONPs being tested. The displayed potential biogenic AgONPs with antibacterial potential is consistent with previous findings^21,42,48^.

**Table 3:** Zone of inhibition values for different bacterial strains.

Bacterial strains	ZOI (μg/ml)
*Bacillus subtilis*	22.5
*Lactobacillus acidophilus*	23
*Staphylococcus aureus*	23.25
*Escherichia coli*	22.25
*Pseudomonas aeruginosa*	22.75
*Rhodococcus jostii*	23.8
*Oxytetracycline*	50

#### Antifungal activity of RC@AgONPs

**Figure 7:**
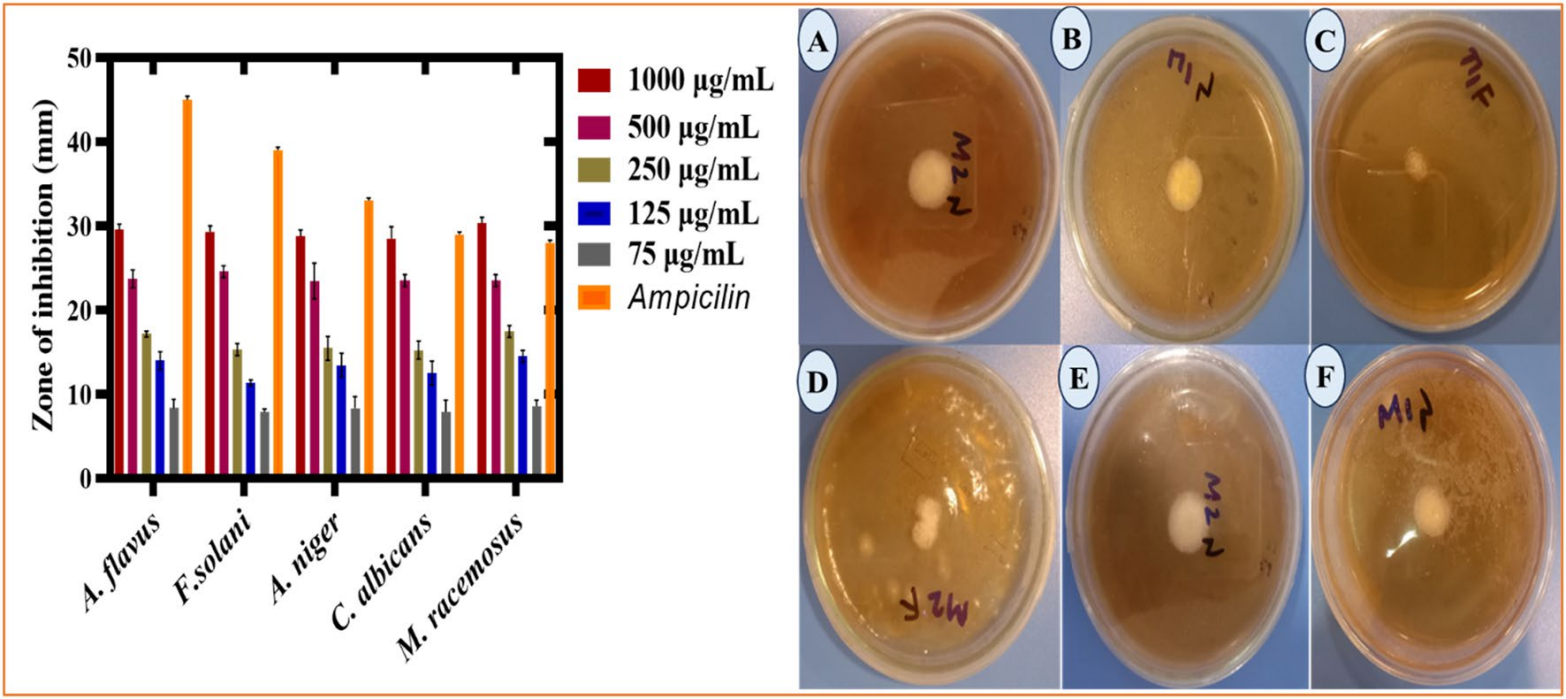
Antifungal potential of biogenic AgONPs at different concentrations.

The antifungal potency of *R*. *capitata*-mediated AgONPs was evaluated against various fungal strains (*Aspergillus niger*, *Aspergillus flavus*, *Candida albicans*, *M. racemosus* and *Fusarium solani*). To the best of our knowledge, no earlier studies have been reported on *R*. *capitata*@ AgONPs. A recent study described the first antifungal activity of AgONPs mediated by *R*. *capitata*. The antifungal activity^26,49^ was determined by employing the disc diffusion method using various AgONPs concentrations (1000–75 μg/mL). *Aspergillus flavus* was detected with a zone of inhibition (ZOI) of 29.5 ± 0.71 mm at a concentration of 1000 µg/mL. Likewise, *A*. *niger* revealed a ZOI of 28.8 ± 0.71 mm at 1000 μg/mL. Furthermore, ZOIs for *M*. *racemosus* (30.31 ± 0.71 mm), *Candida albicans* (28.5 ± 1.41 mm), and *F*. *solani* (29.3 ± 0.71 mm) have been reported (Fig. 7). Nevertheless, not any experimental samples (AgONPs) determined % inhibition more than the drug Amp-B. According to prior investigations, the interaction of AgONPs with the hyphae and spores of fungi resulted in hindering fungal growth due to the production of reactive oxygen species (ROS)^50^. Previous studies^21,41,51^ explained the sufficient dose-dependent antifungal efficiency that is in alignment with our current results (Table 4).

**Table 4:** ZOI values of various fungal strains.

Fungal strains	ZOI (μg/ml)
*Aspergillus flavus*	29.5
*Fusarium solani*	29.3
*Aspergillus niger*	28.8
*Candida albicans*	28.5
*Mucor racemosus*	30.31
*Ampicillin*	48

#### Antihemolytic influences of RC@AgONPs

**Figure 8:**
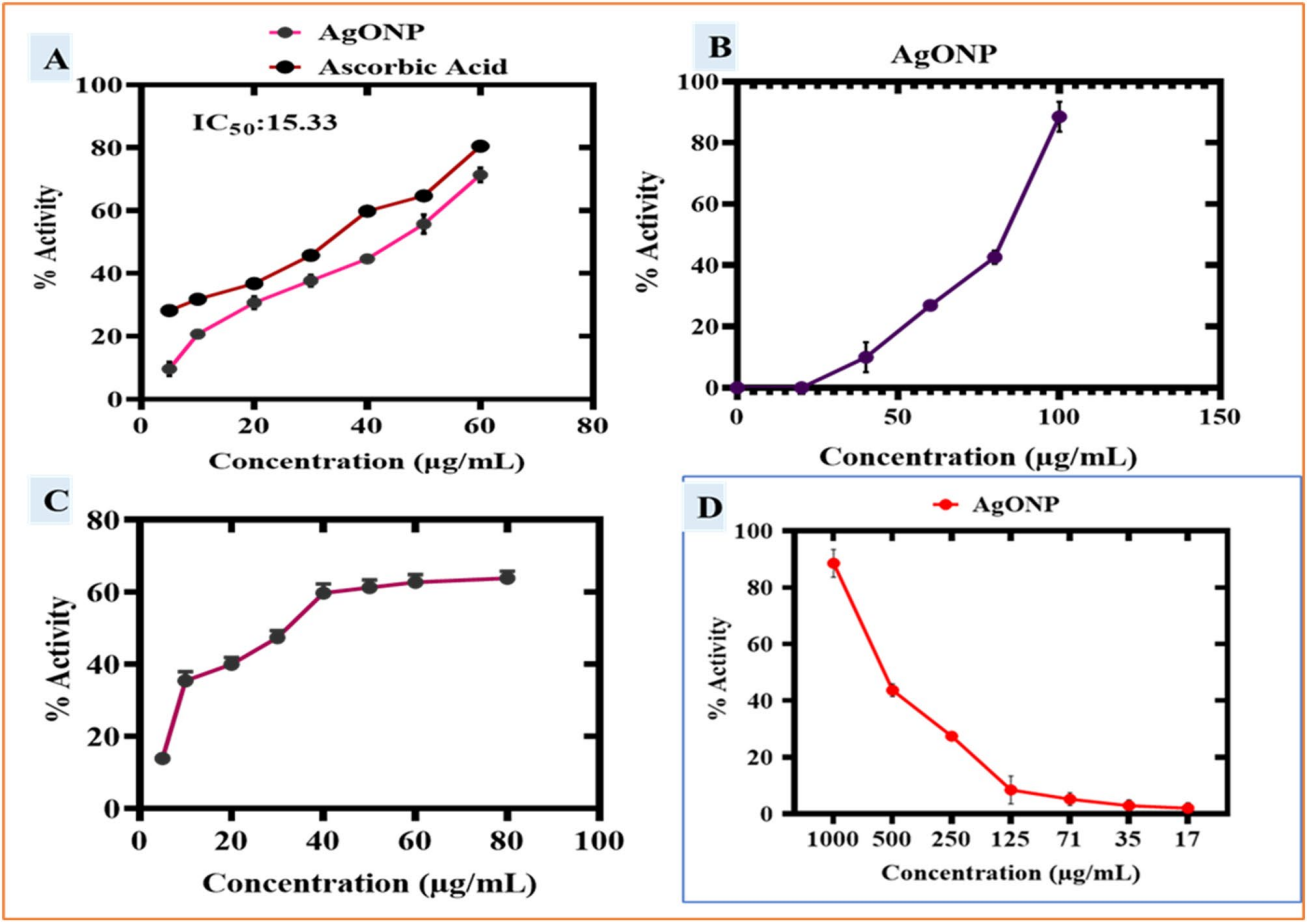
(**A**) Antioxidant (DPPH) potential of *R. capitata-*mediated AgONPs. (**B**) Antioxidant (TAC) potential of *R. capitata-*mediated AgONPs. (**C**) Antioxidant (TRP) potential of AgONPs (**D**)The antihaemolytic potential of *R. capitata-*mediated AgONPs.

An antihemolytic assessment^27^ was determined by treating human red blood cells to verify the safe nature of the synthesized nanoparticles. The biocompatible and toxicological effects of silver oxide NPs were assessed by treating human RBCs. A biological constituent is categorized as hemolytic if it contains a potential level of at least 5%, somewhat hemolytic if it has an activity level of between 2 and 5%, and nonhemolytic if it shows a potential level of less than 2%. Currently, AgONPs have been introduced into RBCs at doses ranging between 1000 and 75 µg/mL. The evidence gained revealed that the produced nanoparticles were nonhemolytic in ranges of 17–71 µg/mL concentrations, slightly hemolytic at doses of 75–125 µg/mL, and found to be more hemolytic at concentrations greater than 125 µg/mL. These results validate prior findings on *Simarouba glauca*, *Rhamnus virgata*, Aristolochia longa, and *Cichorium intybus*-mediated AgONPs^41,42,52,53^. Our study illustrated that the biosynthesized AgONPs are nonhemolytic and considered to be biocompatible at lower concentrations (Fig. 8D), as shown by the biocompatibility assay results of AgONPs.

#### Antioxidant activities of RC@AgONPs

The antioxidative properties (DPPH free radical scavenging, TRP, and TAC) of AgONPs were examined in the current work (Fig. 8A). *R*. *capitata* leaf extract was utilized as a capping, reducing, and oxidizing agent. Numerous phenolic compounds have been found to scavenge ROS, which is connected to the production of synthesized AgONPs. The antioxidant activity was carried out using a dose varying from the highest 1000 µg/mL to the lowest applied 75 µg/mL. At 200 μg/mL AgONPs, the highest total antioxidant capacity (TAC) value was estimated to be 51.4% in terms of AA equivalents per mg. At a concentration of 1000 µg/mL, the value recorded was 87.5 ± 4.8, while at the lowest applied concentration, the value recorded was 6.4 ± 4.7. The TAC scavenging potential of the tested sample RC@AgONPs is shown in (Fig. 8B).

To understand more about the existence of antioxidant species absorbed by AgONPs, a total reducing power assay was examined. This method was used to inspect reductones, which provide hydrogen atoms to the antioxidant capacity and may be susceptible to free radical damage. Biogenic AgONPs have shown considerable TRP efficiency. The reducing power decreased along with the AgONP concentration. The maximum reducing value was 62.68 ± 1.77% at 60 μg/mL, while the lowest value recorded was 13.88 ± 1.36 at a concentration of 5 µg/mL (Fig. 8C). Likewise, at 200 µg/mL, AgONPs demonstrated a substantial capacity to scavenge DPPH radicals (79.4%). Several antioxidant chemicals probably help the *R*. *capitata* leaf extract-induced reduction and stability of AgONPs, as shown by the results in (Fig. 8C). Our findings are consistent with other studies on biogenic AgONPs that were used^21,28,54,55^.

### Biocompatibility assay of RC@AgONPs

**Figure 9:**
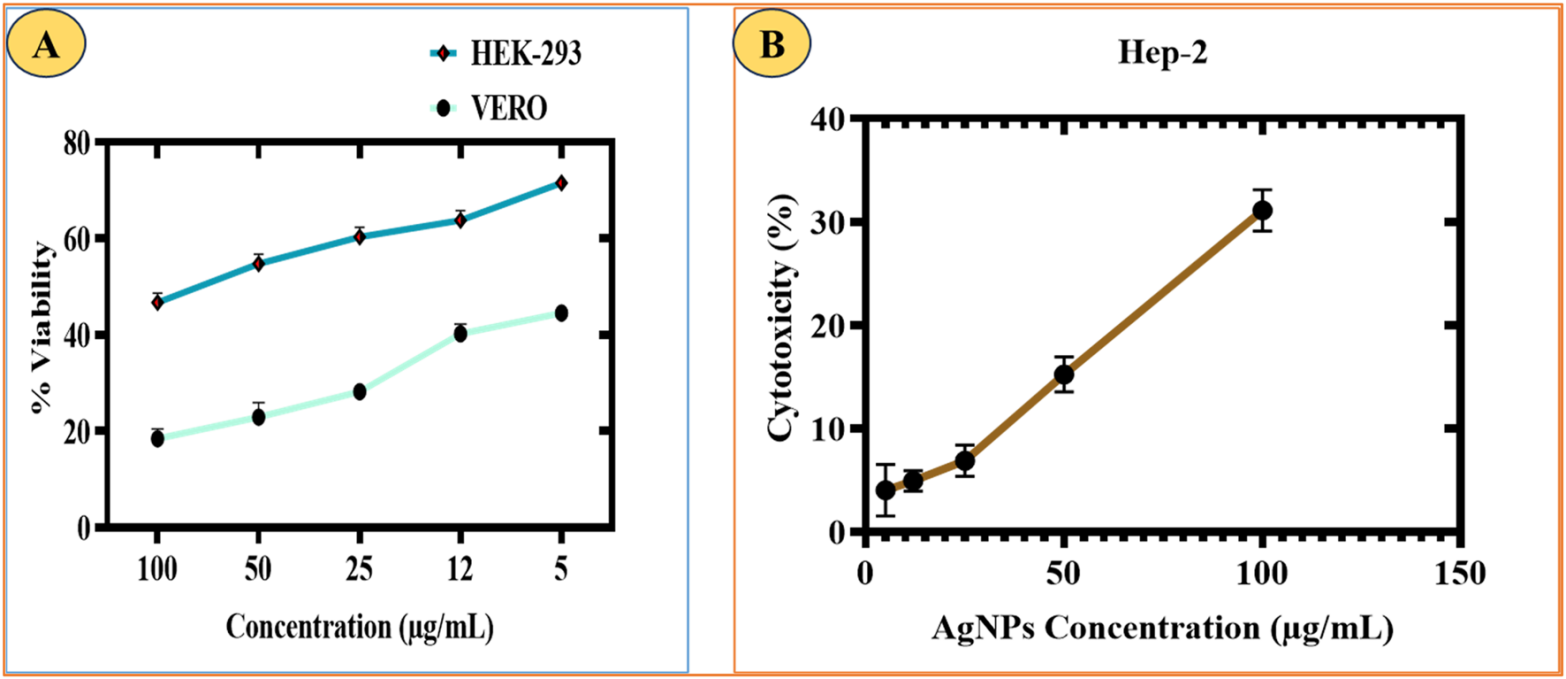
(**A**) Biocompatibility potential of AgONPs against VERO and HEK-293 cell lines. The cell viability (in %) of VERO and HEK-293 cell lines in the presence of various concentrations of AgONPs, doxorubicin (positive control), and untreated cells (negative control) (**B**)Cytotoxic potential of AgONPs against Hep-2 cell lines.

The biocompatibility of RC@AgONPs was confirmed using VERO and HEK-293 cell lines. Both cell lines were seeded in a 96-well plate and grown in DMEM for 24 h to determine their biosafety. Additionally, cells were treated with AgONPs at doses ranging from 100 to 5 µg /mL. To demonstrate the biocompatibility of AgONPs, an MTT cell viability experiment^56^ was carried out. The cell lines reacted to AgONPs in a concentration-dependent manner. The outcomes showed that AgONPs at 100 µg/mL prevent and inhibit the progression and growth of cells by ~ 46%, which determines the biosafe action of biogenic AgONPs. Generally, VERO and HEK-293 cells have shown ways to deal with generated ROS from some external source. Several research findings revealed that ROS are not lethal to VERO or HEK-293 cells at lower concentrations^57,58^. However, if the concentration is above a certain threshold, then these AgONPs are detrimental^59^. The IC_50_ values for AgONPs were estimated as 208.14 µg/mL and 344.90 µg/mL for the VERO and HEK-293 cell lines, respectively (Fig. 9A). Our findings are reliable with other studies on biogenic AgONPs that were used^60,61^.

#### Anticancer activity of RC@AgONPs

Cancer is a deadly disease, and it is anticipated that by 2030, there will be 21 million new cases of the disease^62^. By synthesizing innovative medications with high therapeutic potential, scientists are putting great effort into overcoming this huge menace^63,64^. To determine the anticancer potential of RC@AgONPs, Hep-2 cancer cell lines were treated with different doses (5–100 µg/mL) of RC@AgONPs. Furthermore, 96-well plates were incubated for 24 h to determine the antiproliferative effects of the green-synthesized AgONPs. This led to dose-dependent inhibition of Hep-2 cell viability. The MTT analysis demonstrated that cell viability steadily declined as AgONP dosages increased. The IC_50_ determined for AgONPs was 45.94 μg/mL (Fig. 9B). The synthesized AgONPs have shown strong anticancer activity against Hep-2 cell lines which are reliable with other studies on biogenic AgONPs that were used^30,65–67^.

## Conclusion

The results of this study indicate that biogenic nanoparticles, synthesized using *R*. *capitata* leaf extracts as capping agents, (extensive microscopic and spectroscopic analyses were applied to thoroughly inspect) exhibit promising biological attributes, including notable antioxidant potential, biocompatibility, and confirmed efficiency against cancer and microbial agents. These findings suggest that RC-AgONPs hold substantial potential as pharmaceutical agents for pioneering medicinal development. This study validates the feasibility of biogenic AgONPs as secure and viable alternatives across a broad spectrum of biological applications. As a result, this research holds significant promise for the making of RC-AgONPs-based products relevant to diverse sectors, including biomedicine, biotechnology, pharmaceuticals, and nanotechnology. Furthermore, it presents opportunities in the pursuit of innovative therapies to reduce drug-resistant microbial infections through environmentally sustainable means. Yet, further research is imperative to deepen our understanding of the mechanisms underlying the antioxidant, antibacterial, and anticancer properties exhibited by AgONPs within cellular and biological contexts.

## Statement on guidelines

All experimental studies and experimental materials involved in this research are in full compliance with relevant institutional, national and international guidelines and legislation.

## Data Availability

All the raw data in this research can be obtained from the corresponding authors upon reasonable request.
